# Area distortion under certain classes of quasiconformal mappings

**DOI:** 10.1186/s13660-017-1481-1

**Published:** 2017-09-08

**Authors:** Alfonso Hernández-Montes, Lino F Reséndis O

**Affiliations:** 1 Universidad Autónoma Metropolitana Iztapalapa, San Rafael Atlixco No. 186, Col. Vicentina, Iztapalapa, México, 09340 México; 2 Universidad Autónoma Metropolitana Azcapotzalco, Av. San Pablo No. 180, Col. Reynosa Tamaulipas, Azcapotzalco, México, 02200 México

**Keywords:** 30C62, quasiconformal mapping, area distortion

## Abstract

In this paper we study the hyperbolic and Euclidean area distortion of measurable sets under some classes of *K*-quasiconformal mappings from the upper half-plane and the unit disk onto themselves, respectively.

## Introduction

Let $f:\Omega\to \mathbb {C}$ be an ACL (absolute continuous on lines) homeomorphism in a domain $\Omega\subset \mathbb {C}$ that preserves orientation. If *f* satisfies

$$D_{f}= \frac{\vert f_{z}\vert +\vert f_{\overline{z}}\vert }{\vert f_{z}\vert -\vert f_{\overline{z}}\vert } \leq K \quad \mbox{a.e.} $$

for some $K\geq1$, then *f* is a *K*-quasiconformal mapping, where

$$f_{z} = \frac{1}{2} ( f_{x} -if_{y} ) \quad \mbox{and}\quad f _{\overline{z}} = \frac{1}{2} ( f_{x} +if_{y} ), $$

and $D_{f}$ is called the dilatation of *f*.

In 1956 Beurling and Alhfors solved the boundary value problem for quasiconformal mappings [1]. If $M\geq1$, they gave an explicit formula for the extension of an *M*-quasisymmetric function $h:\mathbb {R}\to \mathbb {R}$ to a quasiconformal mapping $f=u+iv$ from $\mathbb {H}$ onto itself, where $\mathbb {H}$ denotes the upper half-plane. The mapping *f* is called the Beurling-Alhfors extension of *h*. In particular *f* satisfies (see [2])

$$\frac{1}{C y^{2}}\leq\frac{J_{f}(z)}{v^{2}}\leq\frac{C}{y^{2}}, $$

where $J_{f}$ denotes the Jacobian of *f* and $C=C(K)>0$ depends on $K=K(M)$, the maximal dilatation of *f*. Thus, for each measurable subset *E* of $\mathbb {H}$, it holds that

$$\frac{A_{\mathcal{H}}(E)}{C}\leq A_{\mathcal{H}}\bigl(f(E)\bigr) \leq C A_{ \mathcal{H}}(E), $$

where $A_{\mathcal{H}}(\cdot)$ denotes the hyperbolic area in the half-plane $\mathbb {H}$.

In 1994 Astala [3] proved that if *f* is a *K*-quasiconformal mapping from the unit disk $\mathbb {D}$ onto itself, normalized by $f(0)=0$, and if *E* is any measurable subset of the unit disk, then $A_{e}(f(E))\leqslant a(K)A_{e}(E)^{1/K}$, where $A_{e}(\cdot)$ denotes the Euclidean area and $a(K)\to1$ when $K\to1^{+}$.

In 1998 Reséndis and Porter [4] obtained some results about area distortion under quasiconformal mappings on the unit disk $\mathbb {D}$ onto itself with respect to the hyperbolic measure. They also showed the existence of explodable sets; this kind of sets has bounded hyperbolic area, but under a specific quasiconformal mapping its image has infinite hyperbolic area.

In recent years harmonic quasiconformal mappings have been extensively studied, see [5–9] and the papers cited therein. The following two recent results are very close to the results presented in this paper.

In 2007 Knežević and Mateljević [10] proved the following Schwarz-Pick type distortion theorem.

### Theorem 1

*Let* *f* *be a* *K*-*quasiconformal harmonic mapping from the unit disk* $\mathbb {D}$ *onto itself*, *then*

$$\frac{1}{1+k}\frac{1-\vert f(z)\vert ^{2}}{1-\vert z\vert ^{2}}\leq\bigl\vert f_{z}(z)\bigr\vert \leq \frac{1}{1-k}\frac{1-\vert f(z)\vert ^{2}}{1-\vert z\vert ^{2}}, $$

*where* *k* *is defined by* $k:=\frac{K-1}{K+1}$.

In 2012 Min Chen and Xingdi Chen [11] studied the class of $(K,K')$ quasiconformal maps from $\mathbb {H}$ onto itself, and they obtained the following result about area distortion of harmonic mappings.

### Theorem 2

*Let* $f(z)=u(z)+iv(z)$ *be a harmonic mapping from* $\mathbb {H}$ *onto itself and continuous on* $\mathbb {H}\cup \mathbb {R}$ *with* $f(\infty)=\infty$. *In particular*, *f* *has the form* $f(z)=f(x+iy)=u(x,y)+icy$ *for some* $c>0$ (*see* [12]). *If* *f* *is* *K*-*quasiconformal and* $E\subset \mathbb {H}$ *is any measurable set*, *then*

(i) $A_{e}(f(E))\leq c^{2}KA_{e}(E)$,
(ii) $A_{\mathcal{H}}(f(E))\leq K A_{\mathcal{H}}(E)$,
(iii) $0< u_{x}\leq\frac{c(K+1+\sqrt{(K+1)^{2}-4})}{2}$,
(iv) $\vert u_{y} \vert \leq c\sqrt{(K+1)\frac{K+1+ \sqrt{(K+1)^{2}-4}}{2}}$.

In this paper we use the following hyperbolic density definitions:

$$\frac{2\vert dz\vert }{1-\vert z\vert ^{2}}\quad \mbox{and}\quad \frac{ \vert dw\vert }{\operatorname {Im}w} $$

for the unit disk $\mathbb {D}$ and the upper half-plane $\mathbb {H}$, respectively, see [13] and [14]. We denote also by $A_{\mathcal{H}}$ the hyperbolic area in the unit disk $\mathbb {D}$.

## Results and discussion

Our purpose in this article is to continue the study of the hyperbolic area distortion under *K*-quasiconformal mappings from the upper half-plane $\mathbb {H}$ onto itself or from the unit disk $\mathbb {D}$ onto itself. Due to the existence of explodable sets (see [4]), we study some particular classes of quasiconformal mappings. First we apply the result of Knežević and Mateljević [10] to estimate the hyperbolic area distortion under harmonic quasiconformal mappings from $\mathbb {D}$ onto itself, and we remove the hypothesis of harmonicity in Theorem 2.

Additionally, we generalize even more the class studied by Chen and Chen in [11]. More precisely, the main result of this paper is the following.

### Theorem 3

*Let* *f* *be a* *K*-*quasiconformal mapping from* $\mathbb {H}$ *onto itself such that* *f* *maps a family of horocyclics with a common tangent point onto a family of horocyclics*. *Then*, *for each measurable set* $E\subset \mathbb {H}$, *the following inequalities hold*:

$$\frac{1}{K^{9}} A_{\mathcal{H}}(E)\leq A_{\mathcal{H}}\bigl(f(E)\bigr)\leq K ^{9} A_{\mathcal{H}}(E). $$

*These bounds are asymptotically sharp when* $K\to1^{+}$.

Additionally, we obtain some results about radial and angular quasiconformal mappings. Motivated by the generalization mentioned above, we finally describe a set that contains the region of values of the partial derivatives of *K*-quasiconformal mappings.

### Harmonic quasiconformal mappings

In this part we use Theorem 1 to estimate the hyperbolic area distortion under quasiconformal harmonic mappings from the unit disk onto itself and analyze the hyperbolic and Euclidean area distortion under quasiconformal mappings $f(z)=f(x+iy)=u(x,y)+icy$, with $c>0$, from $\mathbb {H}$ onto itself, without the hypothesis of harmonicity of *f* (see [11]), and we sketch the proof of items (i) and (ii) of Theorem 2 as a corollary of this result and get better bounds than the ones obtained in items (iii) and (iv) in the same theorem. See [13] and [14] for results in hyperbolic geometry.

#### Theorem 4

*Let* *f* *be a* *K*-*quasiconformal harmonic mapping from the unit disk* $\mathbb {D}$ *onto itself*. *If* $E\subset \mathbb {D}$ *is a measurable set*, *then*

$$\frac{1}{K}A_{\mathcal{H}}(E)\leq A_{\mathcal{H}}\bigl(f(E)\bigr)\leq \biggl( \frac{K+1}{2} \biggr) ^{2}A_{\mathcal{H}}(E). $$

*These bounds are asymptotically sharp when* $K\to1^{+}$.

#### Proof

Let $w=f(z)=u(z)+iv(z)$, $z\in \mathbb {D}$. By hypothesis and Theorem 1, the mapping *f* satisfies

$$\begin{aligned} \biggl( \frac{1}{1+k}\frac{1-\vert f(z)\vert ^{2}}{1-\vert z\vert ^{2}} \biggr) ^{2} \leq& \bigl\vert f_{z}(z)\bigr\vert ^{2} \\ \leq& \biggl( \frac{1}{1-k}\frac {1-\vert f(z)\vert ^{2}}{1-\vert z\vert ^{2}} \biggr) ^{2}. \end{aligned}$$

Moreover, the Jacobian $J_{f}$ of *f* satisfies

$$\bigl(1-k^{2}\bigr)\vert f_{z}\vert ^{2}\leq J_{f}=\vert f_{z}\vert ^{2}-\vert f_{\overline{z}}\vert ^{2} \leq \vert f_{z}\vert ^{2} . $$

Thus, for each measurable set $E\subset \mathbb {D}$, the following equalities hold:

$$\begin{aligned}& A_{\mathcal{H}}f(E)= \int_{f(E)}\frac{du\,dv}{(1-\vert w\vert ^{2})^{2}}= \int _{E}\frac{J _{f}(z)}{(1-\vert f(z)\vert ^{2})^{2}}\,dx\,dy, \\& \frac{1-k^{2}}{(1+k)^{2}}A_{\mathcal{H}}(E)\leq \int_{E} \frac{J_{f}(z)\,dx\,dy}{(1-\vert f(z)\vert ^{2})^{2}}\leq\frac{1}{(1-k)^{2}}A _{\mathcal{H}}(E) \end{aligned}$$

and the result follows immediately. □

#### Theorem 5

*Let* *f* *be a* *K*-*quasiconformal mapping from the upper half*-*plane* $\mathbb {H}$ *onto itself given by* $f(z)=f(x+iy)=u(x,y)+icy$, *with* $c>0$. *If there exists* $M>0$ *such that* $\vert f_{z}(z)\vert -\vert f_{\overline{z}}(z)\vert \leq M$ *a*.*e*., *then for any measurable subset* *E* *of* $\mathbb {H}$ *the following inequalities hold*:

(i) $A_{e}(f(E))\leq M^{2}K A_{e}(E)$,
(ii) $A_{\mathcal{H}}(f(E))\leq\frac{M^{2}K}{c^{2}}A_{ \mathcal{H}}(E)$.

#### Proof

Since *f* is a *K*-quasiconformal mapping, *f* satisfies $\vert f_{z}\vert + \vert f_{\overline{z}}\vert \leq K (\vert f_{z}\vert -\vert f_{\overline{z}}\vert )$ a.e. in $\mathbb {H}$, and by hypothesis we get $J_{f}=(\vert f_{z}\vert +\vert f_{\overline{z}}\vert ) (\vert f_{z}\vert -\vert f_{\overline{z}}\vert )\leq KM^{2}$ a.e. in $\mathbb {H}$. Then, for any measurable set $E\subset \mathbb {H}$,

$$A_{e}\bigl(f(E)\bigr)= \int_{f(E)}du \,dv= \int_{E}J_{f}\,dx \,dy\leq KM^{2}A_{e}(E) $$

and for the second result we have

$$A_{\mathcal{H}}\bigl(f(E)\bigr)= \int_{f(E)}\frac{du \,dv}{(\operatorname {Im}w)^{2}}= \int _{E}\frac{J_{f}\,dx \,dy}{(cy)^{2}}\leq\frac{M^{2}K}{c^{2}}A_{ \mathcal{H}}(E). $$

 □

If we additionally suppose that *f* is a harmonic mapping, then there exists a holomorphic function $g:\mathbb {H}\to \mathbb {C}$ such that $f(z)= \operatorname {Re}g(z) +icy$. Thus

$$\vert f_{z}\vert =\frac{1}{2} \bigl\vert g'(z)+c\bigr\vert \quad \mbox{and} \quad \vert f_{\overline{z}}\vert = \frac{1}{2} \bigl\vert g'(z)-c\bigr\vert . $$

Since

$$\frac{\vert f_{\overline{z}}\vert }{\vert f_{z}\vert } \leq k \quad \mbox{or equivalently}\quad \bigl\vert g'(z)-c\bigr\vert ^{2} \leq k^{2} \bigl\vert g'(z) +c\bigr\vert ^{2}, $$

we obtain that $g'(z)$ belongs to the circle *D* with center $(\frac{1+k^{2}}{1-k^{2}}c,0)$ and radius $\frac{2ck}{1-k^{2}}$. Hence, for each $w\in D$, we get the estimations

$$\vert w+c\vert -\vert w-c\vert \leq2c\quad \mbox{and}\quad \vert w+c\vert + \vert w-c\vert \leq2Kc. $$

In particular we have

$$\vert f_{z}\vert -\vert f_{\overline{z}}\vert \leq c\quad \mbox{and}\quad \vert f_{z}\vert +\vert f_{\overline{z}}\vert \leq Kc. $$

So we obtain Theorem 2 as a corollary of Theorem 5.

We note that Theorem 5 gives some information about the partial derivatives, in specific we obtain that if *f* satisfies the hypothesis of the last theorem, then $f_{z}$ and $f_{\overline{z}}$ are bounded. The previous sketch gives us also some idea to study quasiconformal mappings of the form $f(x+iy)=u(x,y)+iv(y)$.

### Quasiconformal mappings $f(x+iy)=u(x,y)+iv(y)$

We now generalize the class studied by Cheng and Chen (see [11]) in two directions. First, we will show that it is possible to avoid the harmonic hypothesis, and second, we will prove that the class of *K*-quasiconformal mappings given by $f(x+iy)=u(x,y)+iv(y)$ is a family that accepts asymptotically sharp bi-bounds for the area distortion.

Let $1\leq K<\infty$ and $\Omega\subset \mathbb {C}$ be a domain. Suppose that $f:\Omega\to \mathbb {C}$ is a *K*-quasiconformal mapping given by

1 $$ f(x+iy)=u(x,y)+iv(x,y). $$

Then *f* satisfies

2 $$ \biggl\vert \frac{f_{\overline{z}}}{f_{z}} \biggr\vert \leq k, \quad \mbox{a.e.}\quad \mbox{or equivalently}\quad \biggl\vert \frac{f_{ \overline{z}}}{f_{z}} \biggr\vert ^{2}\leq k^{2},\quad \mbox{a.e.} $$

Inequality (2) is satisfied if and only if

$$(u_{x}-v_{y}) ^{2}+(v_{x}+u_{y})^{2} \leq k ^{2} \bigl((u_{x}+v_{y})^{2}+(v _{x}-u_{y})^{2}\bigr)\quad \mbox{a.e.} $$

or equivalently

$$u_{x}^{2}+u_{y}^{2}+v_{x}^{2}+v_{y}^{2}- \frac{1+k^{2}}{1-k^{2}}2u _{x}v_{y}+\frac{1+k^{2}}{1-k^{2}}2u_{y}v_{x} \leq0 \quad \mbox{a.e.} $$

Define

3 $$ \alpha=\alpha(k):=\frac{1+k^{2}}{1-k^{2}}\geq1. $$

Then *f* satisfies inequality (2) if and only if

4 $$ u_{x}^{2}+u_{y}^{2}+v_{x}^{2}+v_{y}^{2}-2 \alpha u_{x}v_{y}+2\alpha u _{y}v_{x}\leq0 \quad \mbox{a.e.} $$

From now on each expression that involves partial derivatives will be true almost everywhere (a.e.) and Ω will denote a domain of the complex plane $\mathbb {C}$.

In this part we focus on *K*-quasiconformal mappings *f* from $\mathbb {H}$ onto itself given by $f(x+iy)=u(x,y)+iv(y)$. In particular *f* can be extended homeomorphically to $\overline{\mathbb {H}}$, $u(x,y)$ is ACL and $v(y)$ is absolutely continuous. We know that *f* satisfies

5 $$ u_{x}^{2}+u_{y}^{2}+v_{y}^{2}-2 \alpha u_{x}v_{y}\leq0\quad \mbox{a.e.} $$

Despite the fact that *v* depends only on the variable *y*, we write $v_{y}$ instead of $v'$ to emphasize the dependence on *y*. The next result gives the principal characteristics of the mapping *f*, and most of them are consequences of its quasiconformal properties (the rest are easy to prove).

#### Proposition 1

*Let* *f* *be a* *K*-*quasiconformal mapping from* $\mathbb {H}$ *onto itself given by* $f(x+iy)=u(x,y)+iv(y)$. *Then*

- *The function* $y\mapsto v(y)$ *is a homeomorphism from* $[0,\infty) \to[0,\infty)$ *that is absolutely continuous and differentiable a*.*e*.
- *For almost every* $y\in[0,\infty)$, *the function* $x\mapsto u(x,y)$ *is a homeomorphism from* $\mathbb {R}$ *onto itself that is absolutely continuous and differentiable a*.*e*.
- *The* *K*-*quasiconformal inverse mapping* $f^{-1}:\mathbb {H}\to \mathbb {H}$ *has the same form as* *f*, *that is*, $f^{-1}(x+iy)=w(x,y)+iv^{-1}(y)$.
- *Let* $y_{0}\in(0,\infty)$ *be fixed*. *The function* $h:\mathbb {H}\to \mathbb {H}$ *defined by* $h(x+iy)=u(x,y+y_{0})+i[v(y+y_{0})-v(y_{0})]$ *is a* *K*-*quasiconformal mapping*. *In particular each function* $x\mapsto u(x,y _{0})$ *is quasisymmetric*.
- *If* $g:\mathbb {H}\to \mathbb {H}$ *is a* $K'$-*quasiconformal mapping given by* $g(x+iy)=l(x,y)+iw(y)$, *then* $f\circ g$ *is a* $KK'$-*quasiconformal mapping of the same form*.
- *The mapping* *f* *leaves invariant the family of horocyclics with tangential point at infinity*.

We study inequality (5) in more detail. To get this, we complete in (5) the square in $v_{y}$, so we obtain

$$(u_{x}-\alpha v_{y})^{2} + u_{y}^{2} \leq v_{y}^{2}\bigl(\alpha^{2}-1\bigr)\quad \mbox{a.e.} $$

This inequality defines a circle a.e., thus $u_{x}$ and $v_{y}$ satisfy in particular

$$\alpha v_{y}-v_{y}\sqrt{\alpha^{2}-1}\leq u_{x}\leq\alpha v_{y}+ v _{y}\sqrt{ \alpha^{2}-1}\quad \mbox{a.e.} $$

and

$$-v_{y}\sqrt{\alpha^{2}-1}\leq u_{y}\leq v_{y} \sqrt{\alpha^{2}-1}\quad \mbox{a.e.} $$

In fact, the circle is a subset of the square described by the previous inequalities. Observe that

6 $$ K=\alpha+\sqrt{\alpha^{2}-1} $$

and

$$\frac{1}{K}=\alpha-\sqrt{\alpha^{2}-1}, $$

where *K* is the maximal dilatation of *f*. Let $C= \sqrt{\alpha^{2}-1}$. Then $0\leq C$. With this notation the last inequalities can be written as follows:

7 $$ \frac{v_{y}}{K}\leq u_{x}\leq K v_{y} \quad \mbox{a.e.} $$

and

8 $$ -C v_{y}\leq u_{y}\leq Cv_{y} \quad \mbox{a.e.} $$

Given $0\leq x$, we integrate (7) on the interval $[0,x]$

$$\int_{0}^{x}\frac{v_{y}(y)}{K}\,dt\leq \int_{0}^{x}u_{x}(t,y)\,dt\leq \int_{0}^{x} Kv_{y}(y)\,dt. $$

If we choose any fixed $y\in(0,\infty)$ such that $u(x,y)$ is absolutely continuous with respect to *x*, then we obtain

9 $$ \frac{x}{K}v_{y}(y)+u(0,y)\leq u(x,y)\leq Kx v_{y}(y)+u(0,y) $$

for each $x\in \mathbb {R}$ and almost every $y\in(0,\infty)$. Using the left-hand side of the last inequality, we get

$$\limsup_{y\to0^{+}} \biggl[ \frac{v_{y}(y)x}{K}+u(0,y) \biggr] \leq \limsup_{y\to0^{+}}u(x,y), $$

and since $u(x,y)$ is continuous, we obtain

$$\frac{x}{K}\limsup_{y\to0^{+}}v_{y}(y)\leq u(x,0)-u(0,0)< \infty. $$

For this reason, $\limsup_{y\to0^{+}} v_{y}(y)$ exists and consequently $\liminf_{y\to0^{+}}v_{y}(y)$ exists too. We define $v_{y}^{+}(0):= \limsup_{y\to0^{+}}v_{y}(y)$ and $v_{y}^{-}(0):=\liminf_{y\to0^{+}}v _{y}(y)$. With this notation, we obtain from (9)

10 $$ \frac{v_{y}^{+} (0)x}{K}+u(0,0)\leq u(x,0)\leq K v_{y}^{-}(0)x+u(0,0). $$

On the other hand, we choose any fixed $x\in[0,\infty)$ such that $u(x,y)$ is absolutely continuous with respect to *y*, and we integrate (8) on the interval $[0,y]$. So

$$\int_{0}^{y} -Cv_{y}(t)\,dt\leq \int_{0}^{y} u_{y}(x,t)\,dt\leq \int_{0} ^{y} Cv_{y}(t)\,dt $$

and, since $v(y)$ is absolutely continuous, we obtain

$$-Cv(y)+u(x,0)\leq u(x,y)\leq Cv(y)+u(x,0) $$

for $y\in[0,\infty)$ and almost every $x\in[0,\infty)$. By an argument of continuity of the mapping *f* and density, we have

11 $$ -Cv(y)+u(x,0)\leq u(x,y)\leq Cv(y)+u(x,0) $$

for all $(x,y)\in[0,\infty)\times[0,\infty)$. Setting $x=0$ in the previous inequality, we get

12 $$ -Cv(y)+u(0,0)\leq u(0,y)\leq Cv(y)+u(0,0). $$

Thus, combining (9) and (12), we have

$$\frac{v_{y}(y)x}{K}-Cv(y)+u(0,0)\leq u(x,y)\leq Kxv_{y}(y)+Cv(y)+u(0,0) $$

for each $x\in \mathbb {R}$ and almost every $y\in(0,\infty)$. In the same way we use (10) and (11) to obtain

$$\frac{x}{K}v_{y}^{+}(0)-Cv(y)+u(0,0)\leq u(x,y)\leq Kxv_{y}^{-}(0)+Cv(y)+u(0,0). $$

We combine the left- and right-hand sides of the previous inequalities to get

13 $$ \frac{x}{K}v_{y}(y)-Cv(y)+u(0,0)\leq u(x,y)\leq Kxv_{y}^{-}(0)+Cv(y)+u(0,0) $$

and

14 $$ \frac{x}{K}v_{y}^{+}(0)-Cv(y)+u(0,0) \leq u(x,y)\leq Kxv_{y}(y)+Cv(y)+u(0,0) $$

for each $x\in \mathbb {R}$ and almost every $y\in(0,\infty)$. Since the left- and right-hand sides of inequalities (13) and (14) represent linear equations in the variable *x*, we compare their slopes and the fact that $x\geq0$ to conclude

$$\frac{v_{y}(y)}{K}\leq Kv_{y}^{-}(0)\quad \mbox{and}\quad \frac{v_{y}^{+}(0)}{K}\leq Kv_{y}(y) $$

for each $x\in \mathbb {R}$ and almost every $y\in(0,\infty)$. Hence

$$\frac{ v_{y}^{+}(0)}{K^{2}}\leq v_{y}(y)\leq K^{2} v_{y}^{-}(0) $$

for each $x\in \mathbb {R}$ and almost every $y\in(0,\infty)$. We recall that $v(y)$ is absolutely continuous, and we integrate the above inequalities on the interval $[0,y]$

$$\frac{1}{K^{2}} \int_{0}^{y}v_{y}^{+}(0)\,dt\leq \int_{0}^{y}v_{y}(t)\,dt \leq K^{2} \int_{0}^{y}v_{y}^{-}(0)\,dt $$

to get

15 $$ \frac{1}{K^{2}}v_{y}^{+}(0)y\leq v(y)\leq K^{2}v_{y}^{-}(0)y. $$

In particular $0< v_{y}^{-}(0)\leq v_{y}^{+}(0)<\infty$ and *v* has right Dini’s derivatives at 0. If $x<0$, we obtain the same relation as (15) and have the next result.

#### Theorem 6

*Let* *f* *be a* *K*-*quasiconformal mapping from* $\mathbb {H}$ *onto itself given by* $f(x+iy)=u(x,y)+iv(y)$. *Then* $v_{y}^{*}(0)= \limsup_{y\to0^{+}}v_{y}(y)$ *and* $v_{y}^{-}(0)= \liminf_{y\to0^{+}}v_{y}(y)$ *are finite*, *and the partial derivatives of* *f* *satisfy the following inequalities*:

1. $\frac{1}{K^{2}}v_{y}^{+}(0)\leq v_{y}(y)\leq K^{2}v_{y}^{-}(0)$ *for almost every* $y\in(0,\infty)$.
2. $\frac{1}{K^{3}} v_{y}^{+}(0)\leq u_{x}(x,y)\leq K^{3} v_{y}^{-}(0)$ *for almost every* $x+i y\in \mathbb {H}$.
3. $\vert u_{y}(x,y) \vert \leq\frac{K(K^{2}-1)}{2} v_{y}^{-}(0)$ *for almost every* $x+i y\in \mathbb {H}$.

*In particular by Proposition* 6 *the partial derivatives of* *f* *belong to some truncate solid cone*.

We can combine the previous result with items (iii) and (iv) of Theorem 2 to obtain the following result.

#### Corollary 1

*Let* *f* *be a harmonic* *K*-*quasiconformal mapping from* $\mathbb {H}$ *onto itself with* $f(\infty)=\infty$. *In particular there exists* $c> 0$ *such that* $f(x+iy)=u(x,y)+icy$. *Then*

$$\frac{c}{K^{3}}\leq u_{x} \leq A(K)c $$

*and*

$$\vert u_{y} \vert \leq B(K)c $$

*with*

$$\begin{aligned} &A(K)= \textstyle\begin{cases} K^{3} & \textit{if } K\in[1,a], \\ \frac{K+1+\sqrt{(K+1)^{2}-4}}{2} &\textit{if } K\in[a,\infty), \end{cases}\displaystyle \\ &B(K)= \textstyle\begin{cases} \frac{K(K^{2}-1)}{2} & \textit{if } K\in[1,b], \\ \sqrt{(K+1)\frac{K+1+\sqrt{(K+1)^{2}-4}}{2}} &\textit{if } K \in[b,\infty), \end{cases}\displaystyle \end{aligned}$$

*where* $a=1. 12373\ldots$ *and* $b=1. 95371\ldots$ *are the solutions of the equations* $K^{3}= \frac{K+1+\sqrt{(K+1)^{2}-4}}{2}$ *and* $\frac{K(K^{2}-1)}{2}= \sqrt{(K+1)\frac{K+1+\sqrt{(K+1)^{2}-4}}{2}}$, *respectively*.

#### Proof

The left-hand side of the first inequality is immediate from item 1 of Theorem 6. We now consider the right-hand side estimations of $u_{x}$ in item 2 of Theorem 6, with $v_{y}^{-}(0)=v_{y}^{+}(0)=c$ and item (iii) of Theorem 2. Then

$$u_{x} \leq\min \biggl\{ cK^{3}, \frac{c(K+1+\sqrt{(K+1)^{2}-4})}{2} \biggr\} . $$

We consider the equation

$$K^{3}= \frac{K+1+\sqrt{(K+1)^{2}-4}}{2} $$

and obtain the value of *a* by solving $K^{6} -K^{4} -K^{3} +1=0$ that has only two real solutions $K=1$ and $K=1. 12373\ldots=a$.

We now consider the estimations of $\vert u_{y}\vert $ in item 3 of Theorem 6 and item (iv) of Theorem 2. Thus

$$\vert u_{y}\vert \leq\min \biggl\{ \frac{cK(K^{2}-1)}{2}, c \sqrt{(K+1)\frac{K+1+ \sqrt{(K+1)^{2}-4}}{2}} \biggr\} . $$

The value of *b* is given by the real solution of

$$\frac{K(K^{2}-1)}{2}=\sqrt{(K+1) \frac{K+1+\sqrt{(K+1)^{2}-4}}{2}} $$

that can be reduced to the equation

$$\biggl( \frac{K+1}{2} \biggr) \bigl(K^{4}-2K^{3}+K^{2}-2 \bigr)= \sqrt{(K+1)^{2}-4}, $$

and the real solution of this equation is $K=1.95371\ldots=b$. This value is obtained through the equation

$$\biggl( \frac{(K+1)^{2}}{4} \biggr) \bigl(K^{4}-2K^{3}+K^{2}-2 \bigr)^{2}=(K+1)^{2}-4 $$

discarding the value $K=1.55739\ldots$ that obviously is not a solution of the original equation. □

The last corollary shows that these estimations are asymptotically sharp when $K\to1^{+}$ improving the result obtained in [11].

#### Theorem 7

*Let* *f* *be a* *K*-*quasiconformal mapping from* $\mathbb {H}$ *onto itself given by* $f(x+iy)=u(x,y)+iv(y)$. *Then*

1. *There exists* $M>0$ *such that* $\vert f_{z}\vert - \vert f_{\overline{z}}\vert \leq M$ *and* $\vert f_{z}\vert +\vert f_{\overline{z}}\vert \leq KM$ *a*.*e*.
2. *The mapping* *f* *is Lipschitz in* $\mathbb {H}$.
3. *The mapping* *f* *is hyperbolically Lipschitz in* $\mathbb {H}$.

#### Proof

1. Since
  $$\begin{aligned} \vert f_{z}\vert -\vert f_{\overline{z}}\vert &= \frac{1}{2}\sqrt{(u_{x}+v_{y})^{2}+u _{y}^{2}}-\frac{1}{2}\sqrt{(u_{x}-v_{y})^{2}+u_{y}^{2}} \\ &\leq\frac{1}{2}\sqrt{2\bigl(u_{x}^{2}+v_{y}^{2}+u_{y}^{2} \bigr)} \quad \mbox{a.e.} \end{aligned}$$
  We estimate the last expression using Theorem 6 to obtain
  $$\begin{aligned}& \frac{1}{2}\sqrt{2\bigl(u_{x}^{2}+v_{y}^{2}+u_{y}^{2} \bigr)} \\& \quad \leq\frac{\sqrt{2}}{2}\sqrt{\bigl(K^{3} v_{y}^{-}(0)\bigr)^{2}+\bigl(K^{2} v _{y}^{-}(0)\bigr)^{2}+ \biggl( \frac{K^{2}-1}{2}Kv_{y}^{-}(0) \biggr) ^{2}} \\& \quad =\frac{K^{2}v_{y}^{-}(0)}{\sqrt{2}}\sqrt{K^{2}+1+ \biggl( \frac{K ^{2}-1}{2K} \biggr) ^{2}} \\& \quad =\frac{K^{2}v_{y}^{-}(0)}{\sqrt{2}}\sqrt{\frac{5K^{4}+2K^{2}+1}{4K ^{2}}} \\& \quad =\frac{Kv_{y}^{-}(0)}{2\sqrt{2}}\sqrt{5K^{4}+2K^{2}+1} \quad \mbox{a.e.} \end{aligned}$$
  Thus we choose $M=\frac{Kv_{y}^{-}(0)}{2\sqrt{2}}\sqrt{5K^{4}+2K ^{2}+1}$.
2. Let $z_{1}, z_{2}\in \mathbb {H}$ and *l* be the Euclidean segment that joints $z_{1}$ with $z_{2}$. Then
  $$\begin{aligned} \bigl\vert f(z_{1})-f(z_{2})\bigr\vert \leq& \int_{f(l)}\vert df\vert \\ =& \int_{l}\bigl(\vert f_{z}\vert + \vert f_{\overline{z}}\vert \bigr)\vert dz\vert \\ \leq& MK\vert z_{1}-z_{2}\vert . \end{aligned}$$
3. Let $z_{1}, z_{2}\in \mathbb {H}$ and *l* be the hyperbolic segment that joints $z_{1}$ with $z_{2}$. Then
  $$\begin{aligned} d_{\mathcal{H}} \bigl( f(z_{1}),f(z_{2}) \bigr) &\leq \int_{f(l)} \frac{\vert dw\vert }{\operatorname {Im}w}= \int_{l}\frac{\vert df\vert }{v} \\ &\leq \int_{l} \bigl(\vert f_{z}\vert +\vert f_{\overline{z}}\vert \bigr)\frac{\vert dz\vert }{v}\leq MK \int _{l}\frac{\vert dz\vert }{v}\leq LMKd_{\mathcal{H}}(z_{1},z_{2}), \end{aligned}$$
  where $L=\frac{K^{2}}{v_{y}^{+}(0)}$ and $d_{\mathcal{H}}$ denotes the hyperbolic metric. □

Now we can extend even more the result obtained by Min Chen and Xindy Chen [11].

#### Theorem 8

*Let* *f* *be a* *K*-*quasiconformal mapping from* $\mathbb {H}$ *onto itself given by* $f(x+iy)=u(x,y)+iv(y)$. *Then*, *for each measurable set* $E\subset \mathbb {H}$,

1. $\frac{(v_{y}^{+}(0))^{2}}{K^{5}} A_{e}(E)\leq A_{e}(f(E))\leq K ^{5}(v_{y}^{-}(0))^{2} A_{e}(E)$.
2. $\frac{1}{K^{9}} ( \frac {v_{y}^{+}(0)}{v_{y}^{-}(0)} ) ^{2} A _{\mathcal{H}}(E)\leq A_{\mathcal{H}}(f(E))\leq K^{9} ( \frac{v _{y}^{-}(0)}{v_{y}^{+}(0)} ) ^{2} A_{\mathcal{H}}(E)$.

*Since* $v_{y}^{-}(0)\leq v_{y}^{+}(0)$, *we have*

$$\frac{1}{K^{9}} A_{\mathcal{H}}(E)\leq A_{\mathcal{H}}\bigl(f(E)\bigr)\leq K ^{9} A_{\mathcal{H}}(E), $$

*and these inequalities are asymptotically sharp when* $K\to1^{+}$.

#### Proof

Let $E\subset \mathbb {H}$ be a measurable set. The Jacobian of *f* is $J_{f}=u_{x}v_{y}$. By (7) and item 1 of Theorem 6,

$$\frac{(v_{y}^{+}(0))^{2}}{K^{5}}\leq J_{f}\leq K^{5} \bigl(v_{y}^{-}(0)\bigr)^{2}\quad \mbox{a.e}. $$

The Euclidean area of $f(E)$ is

$$\int_{E}J_{f}\,dx\,dy=A_{e}\bigl(f(E)\bigr) $$

and in consequence

$$\frac{(v_{y}^{+}(0))^{2}}{K^{5}} A_{e}(E)\leq A_{e}\bigl(f(E)\bigr)\leq K^{5}\bigl(v _{y}^{-}(0)\bigr)^{2} A_{e}(E). $$

On the other hand, for the hyperbolic area only, we see that from (15) it follows

$$\frac{1}{K^{9}} \biggl( \frac{v_{y}^{+}(0)}{v_{y}^{-}(0)} \biggr) ^{2} A _{\mathcal{H}}(E)\leq A_{\mathcal{H}}\bigl(f(E)\bigr)\leq K^{9} \biggl( \frac{v _{y}^{-}(0)}{v_{y}^{+}(0)} \biggr) ^{2} A_{\mathcal{H}}(E). $$

 □

Now, we can prove Theorem 3.

#### Proof of Theorem 3

If $f:\mathbb {H}\to \mathbb {H}$ is a *K*-quasiconformal mapping that leaves invariant the family of horocyclics with common tangent point at ∞, then $f(x+iy)=u(x,y)+iv(y)$ and is exactly Theorem 8.

We now suppose that $f:\mathbb {H}\to \mathbb {H}$ is a *K*-quasiconformal mapping such that *f* maps horocyclics with tangential point at $x_{0}\in \mathbb {R}$ onto horocyclics with tangential point at $x_{1}\in \mathbb {R}$. For $i=0, 1$, let $H_{i}$ be a horocyclic with tangential point at $x_{i}$ and $H_{\infty}^{i}\subset \mathbb {H}$ be a horocyclic with tangential point at infinity. Then there exist Möbius transformations $T_{i}$ such that $T_{i}(H_{i})=H_{\infty}^{i}$. We define $\hat{f}:H_{\infty}^{0}\to H_{\infty}^{1}$ by $\hat{f}(z)=(T _{1}\circ f\circ T_{0}^{-1})(z)$, then *f̂* is *K*-quasiconformal and can be written in the form $\hat{f}(x+iy)=u(x,y)+iv(y)$. By Theorem 8 we have that if $E\subset \mathbb {H}$ is a measurable set, then *f̂* satisfies

$$\frac{1}{K^{9}} A_{\mathcal{H}}\bigl(T_{0}(E)\bigr)\leq A_{\mathcal{H}}\bigl( \hat{f}\bigl(T_{0}(E)\bigr)\bigr)\leq K^{9} A_{\mathcal{H}}\bigl(T_{0}(E)\bigr). $$

Since the hyperbolic area in $\mathbb {H}$ is invariant under the Möbius transformations $T_{i}$, the result follows immediately. The other case is similar. □

#### Corollary 2

*Let* *f* *be a* *K*-*quasiconformal mapping from* $\mathbb {H}$ *onto itself given by* $f(x+iy)=u(x,y)+iv(y)$. *Then*, *for each measurable set* $E\subset \mathbb {H}$,

(i) *If* *v* *is differentiable at* 0, *then*
  $$\frac{(v'_{y}(0))^{2}}{K^{9}} A_{e}(E)\leq A_{e}\bigl(f(E)\bigr)\leq K^{9} \bigl(v'_{y}(0)\bigr)^{2} A_{e}(E). $$
(ii) *If* *v* *is continuously differentiable in a neighborhood of* 0,
  $$\frac{(v'_{y}(0))^{2}}{K^{5}} A_{e}(E)\leq A_{e}\bigl(f(E)\bigr)\leq K^{5} \bigl(v'_{y}(0)\bigr)^{2} A_{e}(E). $$

*These inequalities are asymptotically sharp when* $K\to1^{+}$.

#### Proof

If *v* is differentiable at 0, then from (15) we get $\frac{v_{y}^{-}(0)}{K^{2}}\leq v_{y}'(0)\leq K^{2} v_{y}^{+}(0)$. If *v* is continuously differentiable in a neighborhood of 0, then $v_{y}^{-}(0)=v_{y}'(0)=v_{y}^{+}(0)$. We prove the corollary applying item 1 of Theorem 8. □

The following examples of quasiconformal mappings are not harmonic, thus we are generalizing the results obtained in [11].

#### Example 1

1. Let $f:\mathbb {H}\to \mathbb {H}$ given by
  $$f(x+iy)=x+\frac{i}{2} \bigl( 2y\arctan y+2\pi y-\ln\bigl(1+y^{2} \bigr) \bigr). $$
  Then *f* is a $\frac{3\pi}{2}$-quasiconformal mapping with
  $$v_{y}^{+}(0)=v_{y}^{-}(0)=v'(0)= \pi. $$
  Thus, for each measurable set $E\subset \mathbb {H}$, we have
  $$\frac{32}{243\pi^{3}}A_{e}(E)\leq A_{e}\bigl(f(E)\bigr)\leq \frac{243\pi^{7}}{32}A_{e}(E) $$
  and
  $$\frac{512}{19{,}683\pi^{9}}A_{\mathcal{H}}(E)\leq A_{\mathcal{H}}\bigl(f(E)\bigr) \leq \frac{19{,}683\pi^{9}}{512}A_{\mathcal{H}}(E). $$
2. Let $f:\mathbb {H}\to \mathbb {H}$ given by $f(x+iy)=2x+\sin(x+y)+iy$. Then *f* is a $\frac{11 +\sqrt{85}}{6}$-quasiconformal mapping with
  $$v_{y}^{+}(0)=v_{y}^{-}(0)=v'(0)=1. $$
  It is enough to observe that the dilatation of *f* is given by
  $$\begin{aligned}& D_{f}(x,y) \\& \quad =\frac{\sqrt{\cos^{2}(x+y)+(3+\cos(x+y))^{2}}+\sqrt{\cos^{2}(x+y)+(1+ \cos(x+y))^{2}}}{\sqrt{\cos^{2}(x+y)+(3+\cos(x+y))^{2}}-\sqrt{ \cos^{2}(x+y)+(1+\cos(x+y))^{2}}} \end{aligned}$$
  and the function $D_{f}(x,a-x)$ depends only on *a*, more precisely
  $$D_{f}(x,a-x)=\frac{\sqrt{\cos^{2}a+(3+\cos a)^{2}}+\sqrt{\cos ^{2}a+(1+\cos a)^{2}}}{\sqrt{\cos^{2}a+(3+\cos a)^{2}}-\sqrt{\cos ^{2}a+(1+\cos a)^{2}}}. $$
  The maximum of $D_{f}(x,a-x)$ is attained at the critical points $a=2l\pi$, $l=0, \pm1, \pm2,\ldots$ and the maximal dilatation of *f* is
  $$\frac{\sqrt{17}+\sqrt{5}}{\sqrt{17}-\sqrt{5}}=\frac{11+ \sqrt{85}}{6}. $$
  Thus, for each measurable set $E\subset \mathbb {H}$, we have
  $$\biggl( \frac{6}{11+\sqrt{85}} \biggr) ^{5}A_{e}(E)\leq A_{e}\bigl(f(E)\bigr) \leq \biggl( \frac{11+\sqrt{85}}{6} \biggr) ^{5}A_{e}(E) $$
  and
  $$\biggl( \frac{6}{11+\sqrt{85}} \biggr) ^{9}A_{\mathcal{H}}(E)\leq A _{\mathcal{H}}\bigl(f(E)\bigr)\leq \biggl( \frac{11+\sqrt{85}}{6} \biggr) ^{9}A _{\mathcal{H}}(E). $$

### Angular and radial quasiconformal mappings

In this part we obtain the results of area distortion for radial and angular mappings. In the case of angular mappings, we use the hyperbolic model of the unit disk $\mathbb{D}$.

#### Proposition 2

*Let* $f:\Omega\to \mathbb {C}$ *be an ACL mapping*. *If* $f(re^{i\theta})= u(re ^{i\theta})+i v(re^{i\theta})$, *then for a*.*e*. *in* Ω

16 $$\begin{aligned} &4\vert f_{z}\vert ^{2} = \biggl( u_{r} + \frac{v_{\theta}}{r} \biggr) ^{2}+ \biggl( v_{r} - \frac{u_{\theta}}{r} \biggr) ^{2}, \end{aligned}$$

17 $$\begin{aligned} &4\vert f_{\overline{z}}\vert ^{2}= \biggl( u_{r} - \frac{v_{\theta}}{r} \biggr) ^{2}+ \biggl( v_{r} + \frac{u_{\theta}}{r} \biggr) ^{2} \end{aligned}$$

*and the Jacobian of* *f* *is*

18 $$ J_{f} =\frac{1}{r}(u_{r}v_{\theta}-u_{\theta}v_{r}). $$

A mapping $f:\mathbb {H}\to \mathbb {H}$ is said to be radial at $x\in \mathbb {R}$ if *f* leaves invariant all Euclidean rays in $\mathbb {H}$ that meet at *x*.

Let $f:\mathbb {H}\to \mathbb {H}$ be a radial mapping at *x*. Since hyperbolic area is invariant under horizontal translations, we can assume that the point $x\in \mathbb {R}$, where the Euclidean rays meet, is $x=0$. If *f* is a radial mapping, then *f* can be written in polar coordinates $(r,\theta)$ as $f(z)=f(re^{i\theta})=\rho(r,\theta)e^{i\theta}$, with $\rho(r, \theta):(0,\infty]\times(0,\pi)\to(0,\infty)$ if $z=re^{i\theta }$.

#### Lemma 1

*Let* *f* *be an ACL mapping from* $\mathbb {H}$ *onto itself*. *Suppose that* *f* *is a radial mapping at* 0. *Then its Jacobian is* $J_{f}=\frac{\rho\rho _{r}}{r}$ *a*.*e*. *If* *f* *preserves orientation*, *then* $\rho_{r}> 0$ *a*.*e*.

#### Proof

Since $f(z)=f(re^{i\theta})=\rho(r,\theta)e^{i\theta}$, then $u(r,\theta)=\rho(r,\theta)\cos\theta$ and $v(r,\theta)=\rho(r, \theta)\sin\theta$, and the proof is immediate from (18). □

#### Proposition 3

*Let* *f* *be a* *K*-*quasiconformal mapping from* $\mathbb {H}$ *onto itself*. *Suppose that* *f* *is a radial mapping at* 0. *Then the function* *ρ* *satisfies the following*:

1. *For* $1\leq r<\infty$,
  $$r^{\frac{1}{K}}\leq\frac{\rho(r,\theta)}{\rho(1,\theta)}\leq r ^{K}; $$
2. *For* $0< r<1$,
  $$r^{K}\leq\frac{\rho(r,\theta)}{\rho(1,\theta)}\leq r^{ \frac{1}{K}}. $$

#### Proof

We first prove that the function $(0,\infty)\ni r\mapsto\ln\rho(r, \theta)$ is absolutely continuous for almost every $\theta\in(0, \pi)$. It is enough to prove that for every $M>1$, the function $[\frac{1}{M},M]\ni r\mapsto\ln\rho(r,\theta)$ is absolutely continuous for almost every $\theta\in(0,\pi)$. Let $\Omega=\{z=x+i \theta\in \mathbb {C}: (x,\theta)\in(-\infty,\infty)\times(0, \pi)\}$. Then the mapping $\log\circ f\circ\exp: \Omega\to \Omega$ is *K*-quasiconformal. Thus the function $(-\infty,\infty) \ni x\mapsto\ln\rho(e^{x},\theta)$ is absolutely continuous for almost every $\theta\in(0,\pi)$. Let $\varepsilon>0$. There exists $\delta>0$ such that for every finite collection of disjoint intervals $(a_{j},b_{j})\subset \mathbb {R}$, $j=1, 2, \ldots, n$, with $\sum_{j=1} ^{n} (b_{j} -a_{j}) < \delta$, then

$$\sum_{j=1}^{n} \bigl( \ln\rho \bigl(e^{b_{j}},\theta\bigr)-\ln\rho\bigl(e^{a_{j}}, \theta\bigr) \bigr) < \varepsilon. $$

Since ln*r* is absolutely continuous on $[\frac{1}{M},M]$, there exists $\delta'>0$ such that for every finite collection of disjoint intervals $(c_{l},d_{l})\subset[\frac{1}{M},M]$, $l=1, 2, \ldots, m$, with $\sum_{l=1}^{m} (d_{l} -c_{l}) < \delta'$, then

$$\sum_{l=1}^{m} (\ln d_{l}-\ln c_{l})< \delta, $$

and by the previous inequality

$$\sum_{l=1}^{m} \bigl( \ln \rho(d_{l},\theta)-\ln\rho(c_{l},\theta) \bigr) < \varepsilon. $$

If $f(z)=\rho(r,\theta)e^{i\theta}$, from (16) and (17) we get

19 $$ \vert f_{z}\vert ^{2}=\frac{1}{4} \biggl( \rho^{2}_{r}+2\frac{\rho_{r}\rho }{r}+\frac{ \rho^{2}}{r^{2}}+ \frac{\rho^{2}_{\theta}}{r^{2}} \biggr)\quad \mbox{a.e.} $$

and

20 $$ \vert f_{\overline{z}}\vert ^{2}=\frac{1}{4} \biggl( \rho_{r}^{2}-\frac{2\rho _{r} \rho}{r}+ \frac{\rho^{2}}{r^{2}}+\frac{\rho_{\theta}^{2}}{r ^{2}} \biggr)\quad \mbox{a.e.} $$

By (2), (19) and (20) we have

$$\frac{1}{4} \biggl[ \biggl( \rho_{r}-\frac{\rho}{r} \biggr) ^{2}+\frac{ \rho^{2}_{\theta}}{r^{2}} \biggr] \leq k^{2} \frac{1}{4} \biggl[ \biggl( \rho _{r}+\frac{\rho}{r} \biggr) ^{2}+\frac{\rho^{2}_{\theta }}{r^{2}} \biggr]\quad \mbox{a.e.} $$

or equivalently

$$\frac{2(r^{2}\rho_{r}^{2}+\rho^{2}+\rho_{\theta}^{2})}{4r\rho_{r} \rho}\leq\frac{k^{2}+1}{1 -k^{2}}=\alpha\quad \mbox{a.e.} $$

Then

$$\frac{1}{2} \biggl[ \frac{r\rho_{r}}{\rho}+\frac{\rho}{r\rho _{r}} \biggr] \leq \alpha\quad \mbox{a.e.} $$

and thus

$$\biggl( \frac{r\rho_{r}}{\rho} \biggr) ^{2}-2\alpha \biggl( \frac {r\rho _{r}}{\rho} \biggr) +1\leq0 \quad \mbox{a.e.} $$

Solving this inequality, we obtain

21 $$ \frac{1}{Kr}\leq\frac{\rho_{r}}{\rho}\leq\frac{K}{r}\quad \mbox{a.e.} $$

or equivalently

$$\frac{1}{Kr}\leq\frac{\partial}{\partial r}\ln\rho\leq \frac{K}{r}\quad \mbox{a.e.} $$

We choose any fixed $\theta\in(0,\pi)$ such that $\ln\rho(r, \theta)$ is absolutely continuous on *r*, and we integrate the previous inequality on the interval $[1,R]$ to get

$$\int_{1}^{R}\frac{1}{Kr}\,dr\leq \int_{1}^{R}\frac{\partial }{\partial r}\ln\rho \,dr\leq \int_{1}^{R}\frac{K}{r}\,dr. $$

Thus

$$\frac{1}{K} \ln r \bigl\vert _{1}^{R}\leq\ln \rho(r,\theta)\bigr\vert _{1}^{R} \leq K \ln r \vert _{1}^{R} \quad \mbox{for almost every }\theta\in(0, \pi)\mbox{ and }R\in[1,\infty). $$

By an argument of continuity of *f* and density, we finally obtain

$$R^{\frac{1}{K}}\leq\frac{\rho(R,\theta)}{\rho(1,\theta)}\leq R ^{K}\quad \mbox{for all }(R, \theta)\in[1,\infty)\times(0,\pi). $$

In a similar way, if we suppose that $0< R<1$, then

$$R^{K}\leq\frac{\rho(R,\theta)}{\rho(1,\theta)}\leq R^{ \frac{1}{K}}\quad \mbox{for all }(R, \theta)\in(0,1)\times(0, \pi). $$

 □

#### Theorem 9

*Let* *f* *be a* *K*-*quasiconformal mapping from* $\mathbb {H}$ *onto itself that leaves invariant each ray in* $\mathbb {H}$ *that meets a real base point*. *If* $E\subset \mathbb {H}$ *is a measurable set*, *then*

$$\frac{1}{K}A_{\mathcal{H}}(E)\leq A_{\mathcal{H}}\bigl(f(E)\bigr)\leq K A_{ \mathcal{H}}(E). $$

*These inequalities are asymptotically sharp when* $K\to1^{+}$.

#### Proof

We can suppose that the base point is 0 since hyperbolic area is invariant under horizontal translations. Let $E\subset \mathbb {H}$ be a measurable set and *Ê* denote the set *E* in polar coordinates. If $f:\mathbb {H}\to \mathbb {H}$ is given by $f(z)=\rho(r,\theta)e ^{i\theta}$, then

$$A_{\mathcal{H}}\bigl(f(E)\bigr)= \iint_{f(E)}\frac{du \,dv}{(\operatorname {Im}w)^{2}}= \iint _{E}\frac{ J_{f}\,dx \,dy}{(\operatorname {Im}f(z))^{2}}= \iint_{\widehat{E}}\frac{ \rho_{r}\,dr \,d\theta}{\rho\sin^{2}\theta}. $$

By (21) we have

$$\frac{1}{Kr}\leq\frac{\rho_{r}}{\rho}\leq\frac{K}{r} \quad \mbox{a.e.} $$

then

$$\frac{1}{K} \iint_{\widehat{E}}\frac{r \,dr \,d\theta}{r^{2}\sin^{2} \theta}\leq \iint_{\widehat{E}}\frac{ \rho_{r}\,dr \,d\theta}{\rho \sin^{2}\theta}\leq K \iint_{\widehat{E}}\frac{r \,dr \,d\theta}{r^{2} \sin^{2}\theta} $$

or equivalently, in rectangular coordinates,

$$\frac{1}{K} \iint_{E}\frac{ dx \,dy}{y^{2}}\leq A_{\mathcal{H}}\bigl(f(E) \bigr) \leq K \iint_{E}\frac{ dx \,dy}{y^{2}}, $$

that is,

$$\frac{1}{K}A_{\mathcal{H}}(E)\leq A_{\mathcal{H}}\bigl(f(E)\bigr)\leq K A_{ \mathcal{H}}(E). $$

 □

#### Example 2

Let $f:\mathbb {H}\to \mathbb {H}$ be the $\frac{\sqrt{2}+1}{\sqrt{2}-1}$-quasiconformal mapping which is radial at 0 given by

$$f\bigl(re^{i\theta}\bigr)=r \biggl( \frac{\theta^{2}}{2}+\theta+1 \biggr) e^{i \theta}. $$

Then, for all measurable set $E\subset \mathbb {H}$, the mapping *f* satisfies

$$\frac{\sqrt{2}-1}{\sqrt{2}+1}A_{\mathcal{H}}(E)\leq A_{ \mathcal{H}}\bigl(f(E)\bigr)\leq \frac{\sqrt{2}+1}{\sqrt{2}-1} A_{ \mathcal{H}}(E). $$

In the following case we consider the hyperbolic model in the unit disk $\mathbb {D}$.

A mapping $f:\mathbb {D}\to \mathbb {D}$ is said to be angular at $0\in \mathbb {D}$ if *f* leaves invariant each circle in $\mathbb {D}$ with center at 0.

An angular mapping *f* at 0 can be written as $f(z)= f(re^{i\theta })=r e ^{i\varphi(r,\theta)}$, where $\varphi:[0,1)\times[0,2 \pi]\to \mathbb {R}$.

#### Lemma 2

*Let* *f* *be an ACL mapping from* $\mathbb {D}$ *onto itself*. *Suppose that* *f* *is angular at* 0. *Then its Jacobian is* $J_{f}=\varphi_{\theta}$. *If* *f* *preserves orientation*, *then* $\varphi_{\theta}> 0$ *a*.*e*.

#### Proof

Since $f(z)=f(re^{i\theta})=re^{i\varphi(r,\theta)}$, then $u(r,\theta)=r\cos\varphi(r,\theta)$ and $v(r,\theta)=r\sin \varphi(r,\theta)$, and the proof is immediate from (18). □

#### Proposition 4

*Let* *f* *be a* *K*-*quasiconformal mapping from* $\mathbb {D}$ *onto itself which is angular at* 0. *Then*

22 $$ \frac{1}{K}\leq\varphi_{\theta}\leq K \quad \textit{a.e. in }[0,1) \times[0,2\pi]. $$

#### Proof

If $f(z)=f(re^{i\theta})=re^{i\varphi(r,\theta)}$, from (16) and (17) we get

23 $$\begin{aligned}& \begin{aligned} &4\vert f_{z}\vert ^{2} =(1+\varphi_{\theta})^{2} +r^{2} \varphi_{r}^{2}\quad \mbox{a.e. } \\ &4\vert f_{\overline{z}}\vert ^{2} =(1-\varphi_{\theta})^{2} +r^{2} \varphi _{r}^{2}\quad \mbox{a.e. } \end{aligned} \end{aligned}$$

Since

$$\frac{(1-\varphi_{\theta})^{2} }{(1+\varphi_{\theta})^{2} }\leq \frac{ \vert f_{\overline{z}}\vert ^{2}}{\vert f_{z}\vert ^{2}}\leq k^{2} \quad \mbox{a.e.}, $$

we get the result. □

#### Corollary 3

*Let* *f* *be a* *K*-*quasiconformal mapping from* $\mathbb {D}$ *onto itself*. *Suppose that* *f* *is angular at* 0. *Then*, *for each* $0< \theta_{1}<\theta _{2}<2\pi$ *and* $r\in(0,1)$, *the following holds*:

$$\frac{\theta_{2}-\theta_{1}}{K}\leq\varphi(r,\theta_{2}) -\varphi (r, \theta_{1})\leq K (\theta_{2}-\theta_{1}). $$

#### Proof

It is immediate integrating (22) and applying the continuity of $\varphi(r,\theta)$ and density. Thus, it is enough to prove that $\theta\mapsto\varphi(r,\theta)$, $\theta\in(0,2 \pi)$, is absolutely continuous for almost every $r\in(0,1)$.

Let $\Omega_{1}=\{z=x+i\theta\in \mathbb {C}: -\infty< x <0 \mbox{ and } 0< \theta<2\pi\}$ and $\Omega_{2}=\{z=x+i\theta\in \mathbb {C}: -\infty< x <0 \mbox{ and } \varphi(r,0)< \theta<2\pi+ \varphi(r,0)\}$. Then the mapping $\log\circ f\circ\exp: \Omega _{1} \to\Omega_{2}$ is *K*-quasiconformal. Thus the function $(0,2\pi)\ni\theta\mapsto\varphi(e^{x},\theta)$ is absolutely continuous for almost every $x\in(-\infty,0)$, or equivalently $\theta\mapsto\varphi(r,\theta)$, $\theta\in(0,2\pi)$, is absolutely continuous for almost every $r\in(0,1)$. □

#### Theorem 10

*Let* *f* *be a* *K*-*quasiconformal mapping from* $\mathbb {D}$ *onto itself which is angular at *0. *If* $E\subset \mathbb {H}$ *is a measurable set*, *then*

$$\frac{1}{K}A_{\mathcal{H}}(E)\leq A_{\mathcal{H}}\bigl(f(E)\bigr)\leq K A_{ \mathcal{H}}(E). $$

*These inequalities are asymptotically sharp when* $K\to1^{+}$.

#### Proof

Let $E\subset \mathbb {D}$ be a measurable set and *Ê* denote the set *E* in polar coordinates. If $f:\mathbb {D}\to \mathbb {D}$ is given as before by $f(z)=re^{i\varphi(r,\theta)}$, then

$$A_{\mathcal{H}}\bigl(f(E)\bigr)= \iint_{f(E)} \frac{4\,du\,dv}{(1-\vert w\vert ^{2})^{2}}= \iint_{E}\frac{ 4J_{f}\,dx \,dy}{(1-\vert f(z)\vert ^{2})^{2}}= \iint_{\widehat{E}}\frac{ 4 \varphi_{\theta} r \,dr \,d\theta}{(1- r^{2})^{2}}. $$

By (22) we have

$$\frac{1}{K} \iint_{\widehat{E}}\frac{4r \,dr\,d\theta}{(1- r^{2})^{2}} \leq \iint_{\widehat{E}}\frac{ 4\varphi_{\theta} r \,dr \,d\theta }{(1- r^{2})^{2}}\leq K \iint_{\widehat{E}}\frac{4r \,dr\,d\theta}{(1- r ^{2})^{2}} $$

or equivalently, in rectangular coordinates,

$$\frac{1}{K} \iint_{E}\frac{ 4\,dx \,dy}{(1-\vert z\vert ^{2})^{2}}\leq A _{\mathcal{H}}\bigl(f(E) \bigr)\leq K \iint_{E}\frac{ 4\,dx \,dy}{(1-\vert z\vert ^{2})^{2}}, $$

and this concludes the proof. □

#### Example 3

Let $f:\mathbb {D}\to \mathbb {D}$ be a mapping given by $f(re^{i\theta})=re^{i \varphi(r,\theta)}$, where $\varphi(r,\theta)= \theta e^{(\theta -2\pi)r}$. Then by (23)

$$\begin{aligned} \frac{\vert f_{z}\vert +\vert f_{\overline{z}}\vert }{\vert f_{z}\vert -\vert f_{\overline{z}}\vert } & \leq\frac{4\vert f_{z}\vert ^{2}}{\vert f_{z}\vert ^{2}-\vert f_{\overline{z}}\vert ^{2}} \\ & =\frac{e ^{2r(\theta-2\pi)}r^{2}\theta^{2}(\theta-2\pi)^{2} +(1+ e^{r( \theta-2\pi)}(1+r\theta))^{2}}{(1+r\theta)e^{r(\theta-2\pi)}} \\ &\leq e^{r(\theta-2\pi)}r^{2}\theta^{2}(\theta-2 \pi)^{2}+\frac{1}{e ^{r(\theta-2\pi)}}+2+(1+r\theta)e^{r(\theta-2\pi)} \\ &\leq\pi^{4}+e^{2\pi r}+2+(1+2\pi) \\ &\leq\pi^{4}+2\pi+3+e^{2\pi}. \end{aligned}$$

Then *f* is a $\pi^{4}+2\pi+3+e^{2\pi}$-quasiconformal mapping. Thus, for each measurable set *E* of the unit disk $\mathbb {D}$, the following inequalities hold:

$$\frac{1}{\pi^{4}+2\pi+3+e^{2\pi}} A_{\mathcal{H}} (E)\leq A_{ \mathcal{H}} \bigl(f(E)\bigr) \leq\bigl(\pi^{4}+2\pi+3+e^{2\pi}\bigr) A_{\mathcal{H}} (E). $$

Numerical evidence says that the maximal dilatation of *f* can be $e^{2\pi}$.

The following example shows that the result of Theorem 10 can not be generalized to radial mappings at 0.

#### Example 4

Let $K\geq1$. Let $f, g:\mathbb {D}\to \mathbb {D}$ be the *K*-quasiconformal mappings given by $f(re^{i\theta}) = r^{\frac{1}{K}} e^{i\theta}$ and $g(re^{i\theta}) = r^{K} e^{i\theta}$. For each $r\in(0,1)$, define $E_{r}=\{ z\in \mathbb {D}: \vert z\vert < r \}$. Then

$$A_{\mathcal{H}}(E_{r})=\frac{4\pi r^{2}}{1-r^{2}} , \qquad A_{\mathcal{H}} \bigl(f(E _{r})\bigr)=\frac{4\pi r^{\frac{2}{K}}}{1-r^{\frac{2}{K}}} ,\qquad A_{ \mathcal{H}}\bigl( g(E_{r})\bigr)=\frac{4\pi r^{2K}}{1-r^{2K}}. $$

Then if $K>1$, there is not $C>0$ such that

$$A_{\mathcal{H}} \bigl(f(E_{r})\bigr)\leq C A_{\mathcal{H}}(E_{r}) \quad \mbox{or}\quad \frac{ A_{\mathcal{H}}(E_{r})}{C} \leq A_{ \mathcal{H}} \bigl(g(E_{r}) \bigr)\quad \mbox{for all }r\in(0,1). $$

### The Beurling-Ahlfors extension

Using the Beurling-Ahlfors (BA) extension, we give explicit examples of quasiconformal mappings of the form $f(x,y)=u(x,y)+iv(x,y)$ and their associated bi-bounds for the hyperbolic area distortion.

More precisely, let $h:\mathbb {R}\to \mathbb {R}$ be an increasing homeomorphism and define its Beurling-Ahlfors extension $f:\overline{\mathbb {R}}\to \overline{\mathbb {R}}$ by $f(x+iy)=u(x,y)+iv(x,y)$, where

$$u(x,y)=\frac{1}{2y} \int_{-y}^{y}h(x+t)\,dt,\qquad v(x,y)=\frac{1}{2y} \int_{0}^{y} \bigl( h(x+t)-h(x-t) \bigr)\,dt. $$

Let $M\geq1$. An increasing homeomorphism $h:\mathbb {R}\to \mathbb {R}$ is *M*-quasisymmetric if

$$\frac{1}{M}\leq\frac{h(x+t)-h(x)}{h(x)-h(x-t)}\leq M $$

for all $x\in \mathbb {R}$ and $t>0$. It is well known that its BA-extension is a $K=K(M)\geq1$ quasiconformal mapping, even more; Ahlfors proved in [2] that this extension is a quasi-isometry, that is, there exists $0< C=C(K)<\infty$ such that

$$\frac{1}{Cy^{2}}\leq\frac{J(z)}{v^{2}}\leq\frac{C}{y^{2}}\quad \mbox{a.e.} $$

#### Example 5

Let $h(x)=x$. Then *h* is a 1-quasisymmetric function and its BA-extension is the 2-quasiconformal and harmonic mapping defined by $f(x+iy)=x+\frac{iy}{2}$. Moreover, for each measurable set $E\subset \mathbb {H}$,

$$A_{e}\bigl(f(E)\bigr)=\frac{1}{2}A_{e}(E) \quad \mbox{and}\quad A_{\mathcal{H}}\bigl(f(E)\bigr)=2A _{\mathcal{H}}(E). $$

#### Example 6

Let $g(x)=x^{3}$. Then *g* is a $7+4\sqrt{3}$-quasisymmetric function, and its BA-extension is the $20. 7872\ldots$ quasiconformal mapping $f(x+iy)=x^{3}+xy^{2}+\frac{i}{4}(6x^{2}y+y^{3})$. In particular $f(x,y)$ does not have bounded derivatives. Moreover,

$$\frac{y^{2} J(x+iy)}{v(x,y)^{2}}= \frac{12(6 x^{4} - 3 x^{2} y^{2} + y^{4})}{(6 x^{2} + y^{2})^{2}}. $$

Setting $y=cx$ in the right-hand side, we get for $x\neq0$

$$r(c)= \frac{12 (6 - 3 c^{2} + c^{4})}{(6 + c^{2})^{2}} $$

and it is easy to see that

$$\frac{3}{4} \leq r(c)< 12\quad \mbox{for each }c\in \mathbb {R}. $$

Thus, for each measurable set $E\subset \mathbb {H}$, we have

$$\frac{3}{4}A_{\mathcal{H}}(E)\leq A_{\mathcal{H}}\bigl(f(E)\bigr) \leq12 A_{ \mathcal{H}}(E). $$

Let

$$E=\biggl\{ (x,y)\in \mathbb {H}: x\in[1,\infty), 0< y\leq\frac{1}{x^{2}}\biggr\} . $$

We have that $A_{e}(E)=1$ and, since $J_{f}(x+iy)=\frac{3}{4}(6x^{4}-3x ^{2}y^{2}+y^{4})$, it holds $A_{e}(f(E))=\infty$. Thus *f* explodes Euclidean area.

#### Example 7

Let $g(x+iy)=2x+\sin(x+y)+iy$. Then *g* is a $\frac{11+\sqrt{85}}{6}$-quasiconformal mapping from $\mathbb {H}$ onto itself. Thus the function $h(x)=2x+\sin x$ is $2. 2\ldots$ -quasisymmetric and its BA-extension is $3. 2\ldots$ -quasiconformal, given by

$$f(x+iy)=2x + \frac{\sin x\sin y}{y}+ \frac{i}{y} \biggl( y^{2} + \cos x -\frac{\cos(x+ y)}{2} -\frac{\cos(x-y)}{2} \biggr). $$

In a forthcoming paper we study more deeply quasi-isometric properties of the BA extension.

### A set that contains the region of values of the partial derivatives of *K*-quasiconformal mappings

In this part we study some particular forms of the mapping *f* in (1). First, suppose that *f* is a *K*-quasiconformal mapping given by $f(x+iy)=u(x)+iv(y)$. Then, by (4), its partial derivatives satisfy the inequality

24 $$ u_{x}^{2}+v_{y}^{2}-2 \alpha u_{x}v_{y}\leq0\quad \mbox{a.e.} $$

Since $\alpha\geq1$, the discriminant of $u_{x}^{2}+v_{y}^{2}-2 \alpha u_{x}v_{y}$ is non-negative and (24) defines the interior of an angular region with the identification $u_{x} \sim x- \mathrm{axis}$ and $v_{y} \sim y-\mathrm{axis}$. Thus we have proved.

#### Theorem 11

*Let* $1\leq K <\infty$. *If* $f:\Omega\to \mathbb {C}$ *is a* *K*-*quasiconformal mapping given by* $f(x+iy)=u(x)+iv(y)$, *then its partial derivatives belong to one of the angular regions defined by* (24).

#### Proof

The proof follows from the fact that the Jacobian of *f* is always positive. □

If *f* is a *K*-quasiconformal mapping given by $f(x+iy)=u(x,y)+iv(y)$, then (4) reduces to

25 $$ u_{x}^{2}+u_{y}^{2}+v_{y}^{2}-2 \alpha u_{x}v_{y}\leq0 \quad \mbox{a.e.} $$

Inequality (25) suggests studying the quadratic form $Q(x,y,w)=x^{2}+y^{2}+w^{2}-2\alpha xw$, whose associated symmetric matrix is

$$N= \left( \begin{matrix}{} 1 & 0 & -\alpha \\ 0 & 1 & 0 \\ -\alpha& 0 & 1 \end{matrix}\right) . $$

#### Proposition 5

*There exists an invertible matrix* *P* *such that* $P^{-1}NP=D$, *where*

$$D= \left( \begin{matrix}{} 1-\alpha& 0 & 0 \\ 0 & 1 & 0 \\ 0 & 0 & 1+\alpha \end{matrix}\right) . $$

#### Proof

The eigenvalues of *N* are $\lambda_{1}=1-\alpha$, $\lambda_{2}=1$ and $\lambda_{3}=1+\alpha$ with eigenvectors $(1,0,1)$, $(0,1,0)$ and $(1,0,-1)$, respectively. After normalization we obtain the basis $\mathcal{B}:=\{(\frac{1}{\sqrt{2}},0,\frac{1}{\sqrt{2}}), (0,1,0), (\frac{1}{\sqrt{2}},0,-\frac{1}{\sqrt{2}})\}$. Set

$$P= \left( \begin{matrix}{} \frac{1}{\sqrt{2}} & 0 & \frac{1}{\sqrt{2}} \\ 0 & 1 & 0 \\ \frac{1}{\sqrt{2}} & 0 & -\frac{1}{\sqrt{2}} \end{matrix}\right) =P^{-1}. $$

A simple calculus ends the proof. □

#### Corollary 4

*The quadratic form* $\widehat{Q}(\widehat{x},\widehat{y},\widehat{w})=(1- \alpha)\widehat{x}^{2}+\widehat{y}^{2}+(1+\alpha)\widehat{w}^{2}$ *represents the quadratic form* $Q(x,y,w)$ *with basis* $\mathcal{B}$, *where*

$$\left( \begin{matrix}{} x \\ y \\ w \end{matrix}\right) = \left( \begin{matrix}{} \frac{1}{\sqrt{2}} & 0 & \frac{1}{\sqrt{2}} \\ 0 & 1 & 0 \\ \frac{1}{\sqrt{2}} & 0 & -\frac{1}{\sqrt{2}} \end{matrix}\right) \left( \begin{matrix}{} \widehat{x} \\ \widehat{y} \\ \widehat{w} \end{matrix}\right) . $$

*In particular* $Q(x,y,w)\leq0$ *if and only if* $\widehat{Q}( \widehat{x},\widehat{y},\widehat{w})\leq0$.

The solution of $\widehat{Q}(\widehat{x},\widehat{y},\widehat {w})=0$ is a double cone if $\alpha>1$. In fact, $\widehat{Q}(\widehat{x}, \widehat{y},\widehat{w})=0$ if and only if

$$(\alpha-1)\widehat{x}^{2}=\widehat{y}^{2}+(1+\alpha) \widehat{w}^{2} $$

or equivalently

$$\widehat{x}^{2}=\frac{\widehat{y}^{2}}{(\alpha-1)}+\frac{(1+\alpha)}{( \alpha-1)} \widehat{w}^{2} $$

and this is the equation of a double elliptic cone in $\mathbb {R}^{3}$ in the basis $\mathcal{B}$.

#### Proposition 6

*Let* $1< K <\infty$. *If* $f:\Omega\to \mathbb {C}$ *is a* *K*-*quasiconformal mapping given by* $f(x+iy)=u(x,y)+iv(y)$, *then its partial derivatives* $u_{x}$, $u_{y}$ *and* $v_{y}$ *belong to one branch of the elliptic cone* (25).

#### Proof

As we saw *f* is *K*-quasiconformal if and only if $u_{x}$, $u_{y}$ and $v_{y}$ satisfy $Q(u_{x},u_{y},v_{y})\leq0$ that describes the solid cone (25). As *f* preserves orientation, then $J_{f}=u _{x}v_{y}>0$ a.e. Since $v_{y}>0$ a.e., then necessarily $u_{x}>0$ a.e., and the result follows. □

We do not study the case $f(x+iy)=u(x,y)+iv(x)$ because this kind of mapping is not a homeomorphism from $\mathbb {H}$ onto itself and in consequence is not quasiconformal.

We consider now the general case, that is, a quasiconformal mapping $f:\Omega\subset \mathbb {C}\to \mathbb {C}$ given by $f(x+iy)=u(x,y)+iv(x,y)$, then by (4) we have that

$$u_{x}^{2}+u_{y}^{2}+v_{x}^{2}+v_{y}^{2}-2 \alpha u_{x}v_{y}+2\alpha v _{x}u_{y}\leq0\quad \mbox{a.e.} $$

In this case we study the quadratic form $Q(x,y,z,w)=x^{2}+y^{2}+z ^{2}+w^{2}-2\alpha xw+2\alpha yz$ with the associated symmetric matrix

$$N= \left( \begin{matrix}{} 1 & 0 & 0 & -\alpha \\ 0 & 1 & \alpha& 0 \\ 0 & \alpha& 1 & 0 \\ -\alpha& 0 & 0 & 1 \end{matrix}\right) . $$

#### Proposition 7

*There exists an invertible matrix* *P* *such that* $P^{-1}NP=D$, *where*

$$D= \left( \begin{matrix}{} 1-\alpha& 0 & 0 & 0 \\ 0 & 1-\alpha& 0 & 0 \\ 0 & 0 & 1+\alpha& 0 \\ 0 & 0 & 0 & 1+\alpha \end{matrix}\right) . $$

#### Proof

The characteristic polynomial of the matrix *N* is $(1-\lambda)^{4}- \alpha^{2}(1-\lambda)^{2}-\alpha^{2}(1-\lambda)^{2}+\alpha^{4}$ with eigenvalues $\lambda_{1}=1+\alpha$, $\lambda_{2}=1-\alpha$, and both with multiplicity two. The eigenvectors of $\lambda_{1}$ are $(1,0,0,-1)$ and $(0,1,1,0)$ and for $\lambda_{2}$ are $(1,0,0,1)$ and $(0,1,-1,0)$. After normalization we obtain the matrix

$$P= \left( \begin{matrix}{} \frac{1}{\sqrt{2}} & 0 & \frac{1}{\sqrt{2}} & 0 \\ 0 & \frac{1}{\sqrt{2}} & 0 & \frac{1}{\sqrt{2}} \\ 0 & -\frac{1}{\sqrt{2}} & 0 & \frac{1}{\sqrt{2}} \\ \frac{1}{\sqrt{2}} & 0 & -\frac{1}{\sqrt{2}} & 0 \end{matrix}\right) $$

with inverse

$$P^{-1}= \left( \begin{matrix}{} \frac{1}{\sqrt{2}} & 0 & 0 & \frac{1}{\sqrt{2}} \\ 0 & \frac{1}{\sqrt{2}} & -\frac{1}{\sqrt{2}} & 0 \\ \frac{1}{\sqrt{2}} & 0 & 0 & -\frac{1}{\sqrt{2}} \\ 0 & \frac{1}{\sqrt{2}} & \frac{1}{\sqrt{2}} & 0 \end{matrix}\right) . $$

Thus

$$P^{-1}NP=D. $$

 □

#### Corollary 5

*The quadratic form*

$$\widehat{Q}(\widehat{x},\widehat{y},\widehat{z},\widehat{w})=(1- \alpha) \widehat{x}^{2}+(1-\alpha)\widehat{y}^{2}+(1+\alpha) \widehat{z}^{2}+(1+\alpha)\widehat{w}^{2} $$

*represents the quadratic form*

$$Q(x,y,z,w)=x^{2}+y^{2}+z^{2}+w^{2}-2 \alpha xw+2\alpha yz $$

*in the basis*

$$\mathcal{C}= \biggl\{ \biggl(\frac{1}{\sqrt{2}},0,0,-\frac{1}{\sqrt {2}} \biggr),\biggl(0,\frac{1}{ \sqrt{2}},\frac{1}{\sqrt{2}},0\biggr),\biggl( \frac{1}{\sqrt{2}},0,0,\frac{1}{ \sqrt{2}}\biggr),\biggl(0,\frac{1}{\sqrt{2}},- \frac{1}{\sqrt{2}},0\biggr) \biggr\} , $$

*where*

$$\left( \begin{matrix}{} x \\ y \\ z \\ w \end{matrix}\right) = \left( \begin{matrix}{} \frac{1}{\sqrt{2}} & 0 & \frac{1}{\sqrt{2}} & 0 \\ 0 & \frac{1}{\sqrt{2}} & 0 & \frac{1}{\sqrt{2}} \\ 0 & -\frac{1}{\sqrt{2}} & 0 & \frac{1}{\sqrt{2}} \\ \frac{1}{\sqrt{2}} & 0 & -\frac{1}{\sqrt{2}} & 0 \end{matrix}\right) \left( \begin{matrix}{} \widehat{x} \\ \widehat{y} \\ \widehat{z} \\ \widehat{w} \end{matrix}\right) . $$

*In particular* $Q(x,y,z,w)\leq0$ *if and only if* $\widehat{Q}( \widehat{x},\widehat{y},\widehat{z},\widehat{w})\leq0$.

#### Proof

We have the relations

$$\left( \begin{matrix}{} x \\ y \\ z \\ w \end{matrix}\right) = \left( \begin{matrix}{} \frac{1}{\sqrt{2}}(\widehat{x}+\widehat{z}) \\ \frac{1}{\sqrt{2}}(\widehat{y}+\widehat{w}) \\ \frac{1}{\sqrt{2}}(\widehat{w}-\widehat{y}) \\ \frac{1}{\sqrt{2}}(\widehat{x}-\widehat{z}) \end{matrix}\right) . $$

Thus

$$\begin{aligned} Q(x,y,z,w) =&x^{2}+y^{2}+z^{2}+w^{2}-2 \alpha xw+2\alpha yz \\ =&\frac{1}{2} ( \widehat{x}+\widehat{z} ) ^{2}+ \frac{1}{2}( \widehat{y}+\widehat{w})^{2}+\frac{1}{2}( \widehat{w}-\widehat{y})^{2}+ \frac{1}{2} ( \widehat{x}- \widehat{z} ) ^{2} \\ &{}-2\alpha \biggl[ \frac{1}{\sqrt{2}} ( \widehat{x}+\widehat {z} ) \biggr] \biggl[ \frac{1}{\sqrt{2}} ( \widehat{x}-\widehat{z} ) \biggr] +2 \alpha \biggl[ \frac{1}{\sqrt{2}} ( \widehat{w}+\widehat {y} ) \biggr] \biggl[ \frac{1}{\sqrt{2}} ( \widehat{w}-\widehat{y} ) \biggr] \\ =&\frac{1}{2}\bigl[\widehat{x}^{2}+2\widehat{x}\widehat{z}+ \widehat{z} ^{2}\bigr]+\frac{1}{2}\bigl[\widehat{y}^{2}+2 \widehat{y}\widehat{w}+\widehat{w} ^{2}\bigr] \\ &{}+\frac{1}{2}\bigl[\widehat{x}^{2}-2\widehat{x}\widehat{z}+ \widehat{z} ^{2}\bigr]+\frac{1}{2}\bigl[\widehat{w}^{2}-2 \widehat{w}\widehat{y}+\widehat{y} ^{2}\bigr] \\ &{}-\alpha\bigl[\widehat{x}^{2}-\widehat{z}^{2}\bigr]+\alpha \bigl[\widehat{w}^{2}- \widehat{y}^{2}\bigr] \\ =&(1-\alpha)\widehat{x}^{2}+(1-\alpha)\widehat{y}^{2}+(1+ \alpha) \widehat{z}^{2}+(1+\alpha)\widehat{w}^{2} \\ =&\widehat{Q}(\widehat{x},\widehat{y},\widehat{z},\widehat{w}); \end{aligned}$$

and this means that $Q(x,y,z,w)\leq0$ if and only if $\widehat{Q}( \widehat{x},\widehat{y},\widehat{z},\widehat{w})\leq0$. □

#### Proposition 8

*Let* $1\leq K <\infty$. *Let* $f:\Omega\to \mathbb {C}$ *be a* *K*-*quasiconformal mapping given by* $f(x+iy)=u(x,y)+iv(x,y)$. *Then its partial derivatives* $u_{x}$, $u_{y}$ *and* $v_{x}$, $v_{y}$ *belong to the solid bounded by* $Q(x,y,z,w)= \widehat{Q}(\widehat{x},\widehat{y},\widehat{z}, \widehat{w})=0$.

## Conclusions

The classes of mappings introduced in this paper have precise geometrical meaning, in particular, the class of quasiconformal mappings $f(z)=u(x,y) +i v(y)$; see, for example, Proposition 1.

As it is showed in Theorems 3, 6, 8, 9, 10 and Corollaries 1 and 2, we have obtained left and right asymptotic bounds for the hyperbolic or Euclidean area distortion. In some previous results only right bounds were known. Moreover, the examples showed that the different classes of mappings defined in the paper are not empty.

We do not know whether the branch of the elliptic cone (25), mentioned in Proposition 6, coincides or not with the region of variation of the partial derivatives of quasiconformal mappings $f(z)=u(x,y)+iv(y)$.
